# Protective immunity elicited by the nematode-conserved As37 recombinant protein against *Ascaris suum* infection

**DOI:** 10.1371/journal.pntd.0008057

**Published:** 2020-02-13

**Authors:** Leroy Versteeg, Junfei Wei, Zhuyun Liu, Brian Keegan, Ricardo T. Fujiwara, Kathryn M. Jones, Oluwatoyin Asojo, Ulrich Strych, Maria Elena Bottazzi, Peter J. Hotez, Bin Zhan

**Affiliations:** 1 National School of Tropical Medicine, Departments of Pediatrics, Baylor College of Medicine, One Baylor Plaza, Houston, Texas, United States of America; 2 Texas Children’s Hospital Center for Vaccine Development, Baylor College of Medicine, Houston, Texas, United States of America; 3 Departamento de Parasitologia, Universidade Federal de Minas Gerais, Belo Horizonte, Brazil; 4 Department of Chemistry and Biochemistry, Hampton University, Hampton, Virginia, United States of America; 5 National School of Tropical Medicine, Departments of Pediatrics and Molecular Virology & Microbiology, Baylor College of Medicine, One Baylor Plaza, Houston, Texas, United States of America; 6 Department of Biology, College of Arts and Sciences, Baylor University, Waco, Texas; George Washington University School of Medicine and Health Sciences, UNITED STATES

## Abstract

**Background:**

*Ascaris lumbricoides* is one of the three major soil-transmitted gastrointestinal helminths (STHs) that infect more than 440 million people in the world, ranking this neglected tropical disease among the most common afflictions of people living in poverty. Children infected with this roundworm suffer from malnutrition, growth stunting as well as cognitive and intellectual deficits. An effective vaccine is urgently needed to complement anthelmintic deworming as a better approach to control helminth infections. As37 is an immunodominant antigen of *Ascaris suum*, a pig roundworm closely related to the human *A*. *lumbricoides* parasite, recognized by protective immune sera from *A*. *suum* infected mice. In this study, the immunogenicity and vaccine efficacy of recombinant As37 were evaluated in a mouse model.

**Methodology/Principal findings:**

As37 was cloned and expressed as a soluble recombinant protein (rAs37) in *Escherichia coli*. The expressed rAs37 was highly recognized by protective immune sera from *A*. *suum* egg-infected mice. Balb/c mice immunized with 25 μg rAs37 formulated with AddaVax^™^ adjuvant showed significant larval worm reduction after challenge with *A*. *suum* infective eggs when compared with a PBS (49.7%) or adjuvant control (48.7%). Protection was associated with mixed Th1/2-type immune responses characterized by high titers of serological IgG1 and IgG2a and stimulation of the production of cytokines IL-4, IL-5, IL-10 and IL-13. In this experiment, the AddaVax^™^ adjuvant induced better protection than the Th1-type adjuvant MPLA (38.9%) and the Th2-type adjuvant Alhydrogel (40.7%). Sequence analysis revealed that As37 is a member of the immunoglobulin superfamily (IgSF) and highly conserved in other human STHs. Anti-As37 antibodies strongly recognized homologs in hookworms (*Necator americanus*, *Ancylostoma ceylanicum*, *A*. *caninum*) and in the whipworm *Trichuris muris*, but there was no cross-reaction with human spleen tissue extracts. These results suggest that the nematode-conserved As37 could serve as a pan-helminth vaccine antigen to prevent all STH infections without cross-reaction with human IgSF molecules.

**Conclusions/Significance:**

As37 is an *A*. *suum* expressed immunodominant antigen that elicited significant protective immunity in mice when formulated with AddaVax^™^. As37 is highly conserved in other STHs, but not in humans, suggesting it could be further developed as a pan-helminth vaccine against STH co-infections.

## Author summary

*Ascaris* infection is the most common infection of humans living in poverty worldwide and can result in malnutrition and stunted physical and mental development in children. A preventive vaccine is urgently needed as a complementary approach to anthelmintic deworming to increase the efficiency of STH infection control. To develop a vaccine against *Ascaris* infection, an immunodominant antigen, As37 of *A*. *suum*, was cloned and expressed as a soluble recombinant protein in *E*. *coli*. The recombinant As37 protein (rAs37) was highly recognized by protective immune sera from *A*. *suum* infected mice. Balb/c mice immunized with 25 μg rAs37 formulated with the adjuvant AddaVax^™^ showed significant larval worm reduction against challenge with *A*. *suum* infective eggs when compared to a PBS (49.7%) or adjuvant control (48.7%). Protection was associated with a mixed Th1/2-type immune response characterized by high titers of serological IgG1 and IgG2a and stimulation of the production of cytokines IL-4, IL-5, IL-10 and IL-13. The AddaVax^™^ adjuvant induced better protection than the Th1-type adjuvant MPLA (38.9%) and the Th2-type adjuvant Alhydrogel (40.7%). Sequence analysis revealed that As37 was a member of the immunoglobulin superfamily (IgSF) and highly conserved in other human STHs. Anti-As37 antibodies strongly recognized homologs in hookworms (*Necator americanus*, *A*. *ceylanicum*, *A*. *caninum*) and in the whipworm *T*. *muris*, but there was no cross-reaction with human spleen tissue extracts. These results indicate that the nematode-conserved As37 protein could be developed as a pan-helminth vaccine antigen to prevent all STH infections without reacting with human IgSF molecules.

## Introduction

*Ascaris lumbricoides* is one of the three major soil-transmitted gastrointestinal helminths (STHs) that infect more than 440 million people worldwide, commonly in developing countries [1], ranking this neglected tropical disease (NTD) among the most common afflictions of people living in poverty [2]. Children harbor the largest number of this intestinal roundworms, often co-infected with other STHs, such as hookworm and whipworm (*Trichuris trichiura*), causing malnutrition and growth stunting as well as cognitive and intellectual deficits [3,4]. With the Global Burden of Disease study estimating that more than 30 million people suffer from *Ascaris* related complications annually [1], children particularly can experience acute intestinal obstruction and other sequelae.

Despite global efforts led by the World Health Organization (WHO) to make therapeutic anthelminthic drugs available to everyone at risk, in 2018, of the 762.9 million school-aged children and 310.2 million pre-school-aged children who required regular deworming, only 59.4% and 38.2%, respectively were actually treated [5]. Moreover, deworming alone is not sufficient to achieve the elimination of roundworms and other STH infections due to high rates of post-treatment reinfection [6], potential drug resistance in areas with repeated deworming treatment [7–9] and poor access to safe water, sanitation, and hygiene (WASH) [10]. Based on these concerns, a study of almost 400 experts on NTDs concluded that the current approach to deworming using anthelmintic treatment alone will not lead to the elimination of the STH infections and that new technologies will be required in order to achieve the targets of the 2012 London Declaration on NTDs [11].

The development of a preventive vaccine targeting children before exposure to helminths or as part of programs linked to deworming (vaccine-linked chemotherapy to prevent helminth reinfection) would represent a key technology for shaping global STH control and elimination strategies [12]. In addition, due to the common occurrence of co-infections with STHs in the same endemic area, it is desirable to develop a multivalent pan-anthelminthic vaccine targeting all three major helminths [13]. In an effort to develop a vaccine against *Ascaris* infection, it was demonstrated in the early 1980s that pigs immunized with irradiated *A*. *suum* larvae were protected significantly against challenge with *A*. *suum* infective eggs, showing reduced numbers of migrating larvae and adult worms in the intestine [14,15]. Moreover, the anthelmintic-terminated infection at the larval migration stage also induced strong protection in pigs with fewer larvae migrating to the lungs and reduced liver milk-spot damage upon new egg challenge [16]. Furthermore, immunizations with crude extracts of larvae [15] or adult worms [17] also induced strong protection against challenge with *A*. *suum* infective eggs. This immunity was even passively transferable to naïve guinea-pigs via sera [18] and is related to IgG antibodies [17]. Several larval antigens recognized by the protective sera have been identified and many of them induced protective immunity in the immunized animals [19–23]. One of the antigens recognized by protective swine serum is As37, a 37 kDa immunodominant protein containing several immunoglobulin domains [21]. Immunolocalization studies with anti-As37 serum found that the protein was present in both larval and adult stages of the worm, mostly localized in muscle and hypodermic structures [21]. However, it was unknown whether As37 would be able to induce protective immunity in mice. In this study, mice were therefore immunized with recombinant As37 protein (rAs37) formulated with several types of adjuvants. Protective immunity was evaluated against a challenge with *A*. *suum* infective eggs. Sequence homology analysis revealed that As37 is a conserved nematode antigen with high sequence identity to homologs in the other major STHs, including human hookworm (*Necator americanus*, *Ancylostoma duodenale*) and pinworm *Trichuris trichiura*, indicating its suitability as a candidate for a pan-helminth vaccine.

## Materials and methods

### *A*. *suum* parasite and mouse model

*A*. *suum* eggs were collected from the uterus of adult female worms from an infected pig in Brazil and maintained in 0.2 N H_2_SO_4_ until developed to the embryonated infective stage. The embryonated infective eggs were used to orally infect BALB/c mice as previously described [24]. *A*. *suum* larvae hatch in the intestine of mice and then migrate to the liver and lungs. The number of larvae recovered from mouse lung tissue eight days post-infection was used as a correlate for vaccine efficacy [24,25].

### Sequence alignment and analysis

Sequences of As37 and its homologs in other nematodes were collected from GenBank and aligned with Clustal Omega (https://www.ebi.ac.uk/Tools/msa/clustalo/). The secondary structural features are illustrated in a structural homology model of As37 using ESPript [26]. The secondary structure elements were generated by homology modeling with either Phyre2 [27,28] or Swiss model [29]. Phylogenetic trees were generated for those functional protein homologs identified in this study using Phylogeny.fr [30].

### Production of recombinant As37 protein

DNA coding for the full-length As37 was synthesized by GenScript (Piscataway, NJ) and then subcloned into the prokaryotic expression vector pET41a using NdeI and NotI sites to exclude the GST tag. The recombinant As37 protein (rAs37) was expressed in *E*. *coli* BL21(DE3) under induction with 1 mM IPTG. Soluble rAs37 with a hexahistidine-tag at its C-terminus was purified by immobilized metal ion affinity chromatography (IMAC), as described previously [31]. Contaminating endotoxin was removed by anion exchange chromatography with a Hitrap Q HP column (GE Healthcare, Chicago, IL), while the purity of the recombinant protein was determined by SDS–PAGE and the protein concentration was measured using A280 absorption. Endotoxin clearance was confirmed using the Charles River Endosafe-PTS system (Charles River, Houston, TX).

### Immunization and challenge infection

Six-week-old female BALB/c mice were purchased from Taconic and divided into 9 groups with 20 mice each. Three groups of mice were each subcutaneously immunized with 25 μg rAs37 formulated with either 200 μg Alhydrogel (Brenntag, Mülheim, Germany), 20 μg MPLA (InvivoGen, San Diego, CA) or 50 μl AddaVax (50/50, v/v) (InvivoGen, San Diego, CA) in a total volume of 100 μl per mouse. One group of 20 mice was immunized with rAs16, another *A*. *suum* secreted protein which induces protective immunity [25], formulated with Alhydrogel as the positive control. To achieve acquired immunity by infection as a positive control, one group of mice was each repeatedly infected with 1,000 embryonated *A*. *suum* eggs by oral gavage three times on days 1, 21 and 35. The same number of mice was injected with just PBS or adjuvant as negative controls. All immunized mice were boosted twice with the same dose on days 21 and 35. Two weeks after the final immunization, 5 mice from each group were sacrificed, and blood and splenocytes were harvested for immunological assessment. The remaining 15 mice from each group were orally challenged with 2,500 *A*. *suum* embryonated eggs in a total volume of 100 μl. Eight days after infection, all infected mice were sacrificed, lungs were collected and *A*. *suum* lung-stage larvae were harvested using a Baermann apparatus, as previously described [24]. Reduction in lung larval burden was calculated in each immunized group by comparing the number of collected larvae to that in the PBS and adjuvant-only control groups.

### Antibody assay

Sera from all blood samples were isolated and frozen at -20 °C. The serum samples were measured for rAs37-specific IgG isotypes (IgG1, IgG2a) using a modified indirect enzyme-linked immunosorbent assay (ELISA). Briefly, 96-well Nunc-Immuno Maxisorp plates (Thermo Scientific, Waltham, MA) were coated with 100 μL rAs37 at a concentration of 0.781 μg/ml in coating buffer (KPL, Milford, MA) overnight at 4 °C. The coated plates were then blocked overnight with 0.1% BSA in PBST (PBS + 0.05% Tween-20), then incubated with serum samples diluted in 0.1% BSA in PBST. After a 2-hour incubation at room temperature, plates were washed 4 times with PBST, then incubated with 1:4000 diluted horseradish peroxidase (HRP)-conjugated goat anti-mouse IgG1 or IgG2a (Lifespan Biosciences, Seattle WA) for 1 hour at room temperature. The plates were washed 5 times and subsequently, 100 μL of 4° C Sure Blue TMB (KPL, Milford, MA) was added per well. The reaction was terminated by adding 100 μL 1 M HCl. The absorbance was measured at 450 nm using a spectrophotometer (BioTek, Winooski, VT).

### Western blot analysis

To determine the presence of native As37 in *A*. *suum* and its homologs in other nematodes, crude extracts of *A*. *suum* lung-stage larvae were prepared by homogenizing the larvae collected from lungs of infected mice, and the excretory/secretory (ES) products of *A*. *suum* lung larvae were prepared by culturing the live lung larvae in RPMI 1640 medium for 24 hours. Extracts of hookworms *N*. *americanus*, *A*. *ceylanicum*, and *A*. *caninum* were prepared by homogenizing the adult worms maintained in different animal models [32–34]. To determine the possible cross-reaction of As37 with human tissue, human spleen tissue lysate was purchased from Leinco Technologies (Fenton, MO). The recombinant proteins and the worm crude extracts were separated by SDS–PAGE, and then transferred onto PVDF membranes (ThermoFisher, Waltham, MA). After blocking with 5% (w/v) skim milk in PBST, the membrane was incubated with sera from mice immunized with rAs37. Pre-immune mouse sera were used as a negative control. HRP-conjugated goat anti-mouse IgG (Invitrogen, Carlsbad, CA) was used as the secondary antibody. The antibody recognized bands were developed by ECL (GE Healthcare, Chicago, IL). The same amount of BSA was used as a non-relevant negative control.

### Analysis of cytokines secreted by splenocytes upon re-stimulation

Spleens were obtained from mice two weeks after the third immunization and the splenocytes were collected by disassociating the spleens using 100 μm cell strainers. The cells were then washed and suspended in complete RPMI media containing 10% heat-inactivated FBS and 1x pen/strep solution. After being centrifuged at 300 x g for 5 min, the splenocytes were resuspended in 2 mL ACK lysis buffer (Thermo Scientific, Waltham, MA) for 5 min to lyse the red blood cells. After being washed with PBS, the splenocytes were resuspended in complete RPMI media containing 10% DMSO and stored in liquid nitrogen until use.

For the cytokine re-stimulation assay, splenocytes were thawed in a 37 °C water bath and transferred to 5 mL pre-warmed complete RPMI media. Splenocytes were washed once to remove residual DMSO and then seeded in a 96-well U-bottom culture plate (Falcon Corning, Tewksbury, MA) at 1x10^6^ splenocytes per well in 250 μL complete RPMI media and re-stimulated with 25 μg/mL rAs37 at 37 °C, 5% CO_2_ for 72 hours. Positive controls were stimulated with 20 ng/mL PMA and 1 μg/mL ionomycin. Unstimulated negative control cultures were processed concurrently. After 72 hours, splenocytes were pelleted by centrifugation (300 x g for 5 min) and the supernatants were collected for measuring cytokine production. The supernatant samples were tested for IL-4, IL-5, IL-6, IL-10, IL-13, IL-17a, IFN-γ and TNF-α using a Bio-Plex Pro Mouse Th1/Th2 8-plex kit (Bio-Rad, Hercules, CA). To save material and costs, and to increase the sensitivity of the experiment, the kit was used in combination with DA-Bead plates (Curiox Biosystems, Singapore), as previously described [35]. Samples were run on a Bio-Plex Magpix multiplex reader according to the manufacturer’s recommendations (Luminex, Austin, TX). Raw Luminex data were analyzed using the Bio-Plex Manager 6.0 software and plotted in GraphPad Prism 6.0. Signal background in blank media was subtracted from re-stimulated samples.

### Statistical analysis

All data were compared by analysis of variance (ANOVA) using SPSS 13.0 software. Data were expressed as means ± standard deviation. A *p*-value of less than 0.05 was considered statistically significant. The IgG1/IgG2a titer ratio results were analyzed using a Mann-Whitney test using Prism 6 (Graphpad).

### Ethics statement

All studies were approved by the Institutional Animal Care and Use Committee of Baylor College of Medicine (Protocol AN-6297), Assurance numbers D16-00475 (current) and 3823–01 (previous) and were performed in strict compliance with the Guide for the Care and Use of Laboratory Animals (8^th^ Edition) [36].

## Results

### Immunological recognition of As37 by sera from infected mouse with acquired immunity against *A*. *suum* infection

**Fig 1:**
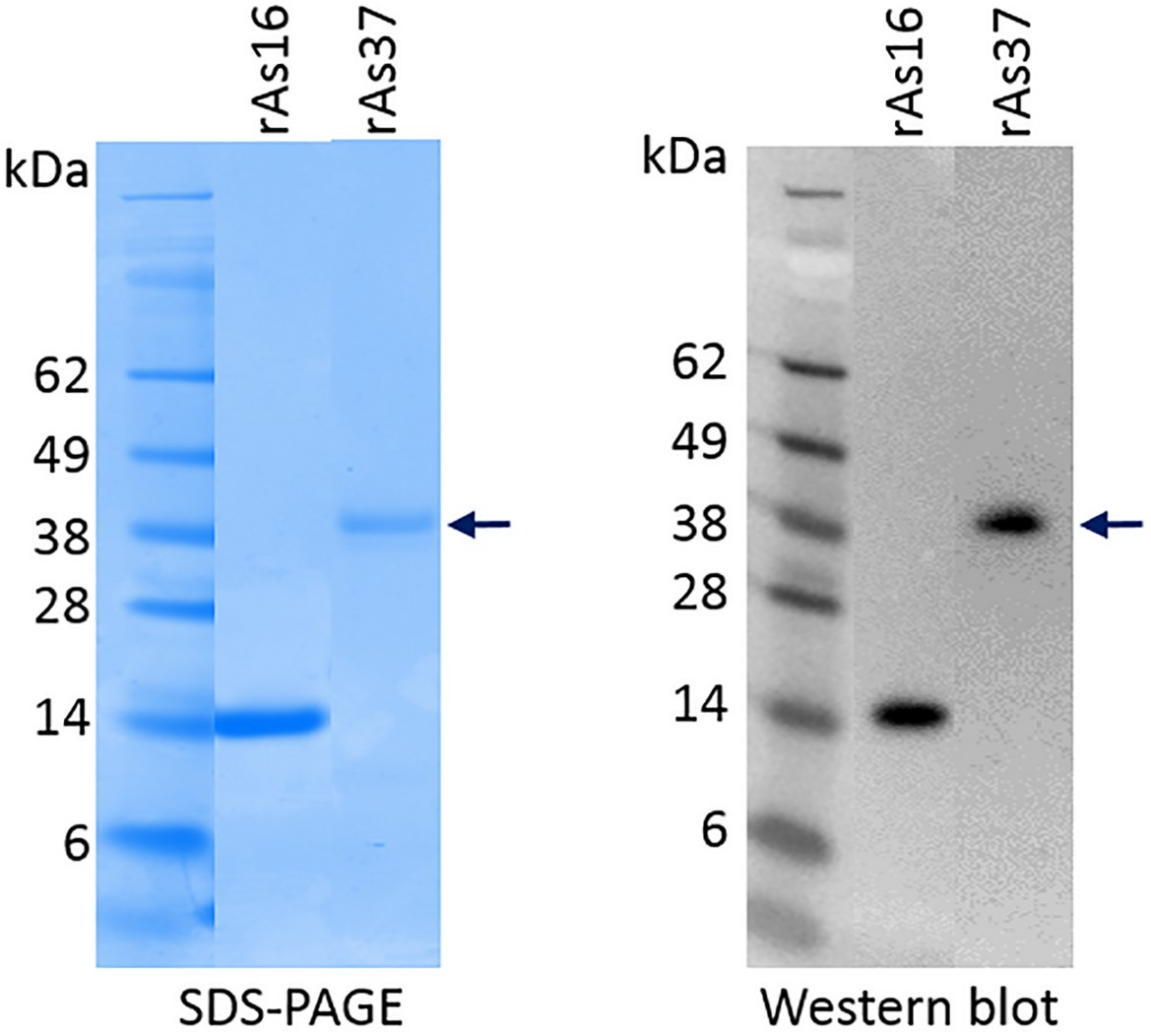
SDS-PAGE of recombinant As16 and As37 expressed in *E*. *coli* (2 μg) and Western blot of recombinant proteins (50 ng) recognized by sera from mice infected with *A*. *suum* eggs (diluted 1:3,000).

Full-length As37 was expressed as a soluble recombinant protein (rAs37) in *E*. *coli* BL21 under induction of 1 mM IPTG. The purified rAs37 (with His-tag) appeared at approximately 38 kDa on SDS-PAGE gels, the size expected based on the sequence. The rAs37, as well as rAs16 that previously had conferred protective immunity in immunized mice [25,37], were both strongly recognized by sera from mice with protective immunity induced by the infection of *A*. *suum* eggs [25] (Fig 1). These results indicate that both As37 and As16 are antigens exposed to the host immune system during *A*. *suum* egg infection and larval migration, making them suitable for their evaluation as vaccine candidates.

### As37 is a highly conserved nematode protein

**Fig 2:**
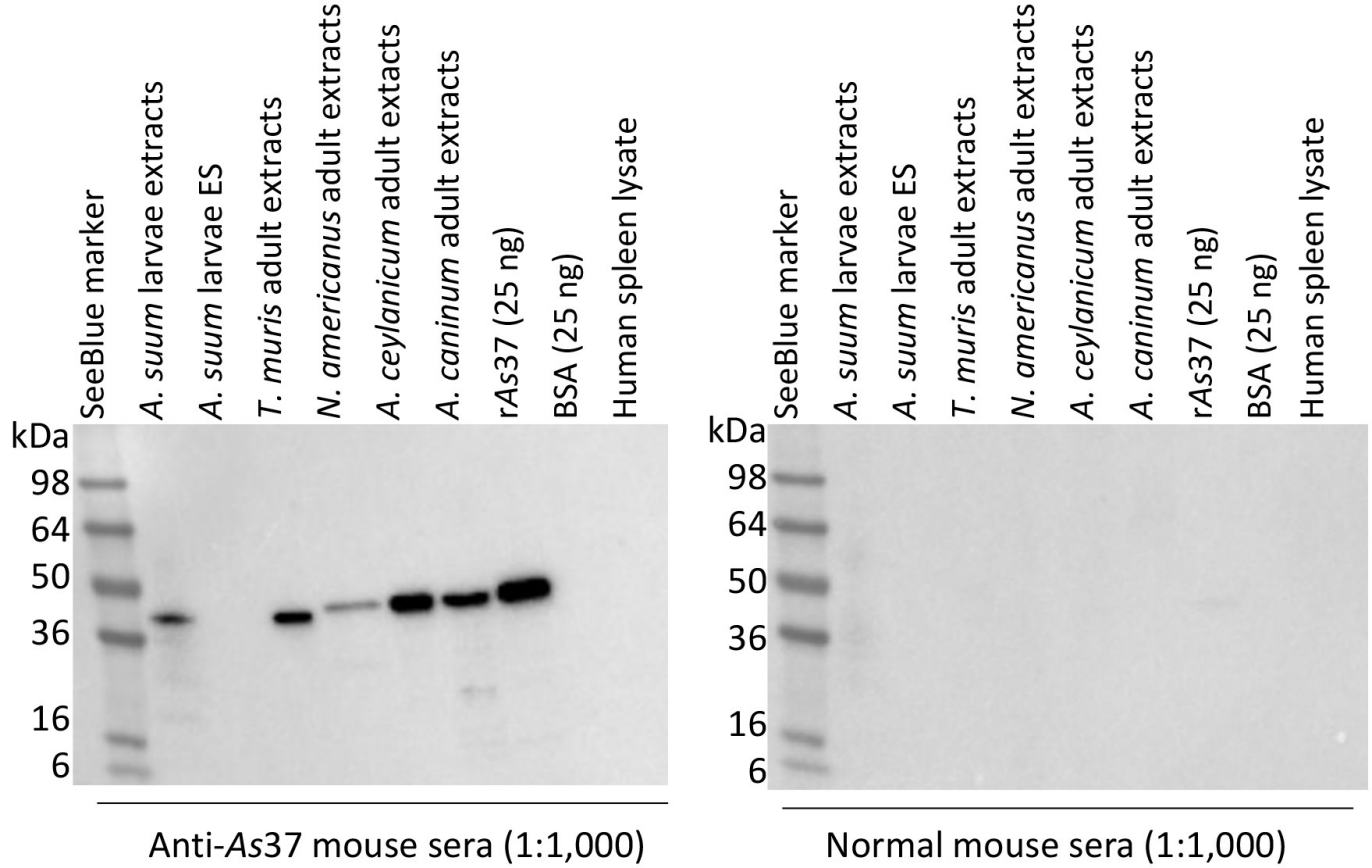
Western blot analysis showing the recognition of native As37 in *A*. *suum* lung larval extracts (10 μg) and its homologs in other nematode extracts (10 μg) by anti-As37 mouse sera (1:1,000) (left), but not by normal mouse serum at the same dilution (right). SeeBlue prestained proteins used as a protein marker. The antisera strongly recognized rAs37 (25 ng), but not BSA (25 ng) and human spleen lysate (10 μg).

Evaluation by Western blot with mouse anti-rAs37 sera demonstrated that anti-rAs37 antisera recognized the native As37 in extracts of *A*. *suum* lung larvae, but not in the ES products of the larvae. The antisera further recognized As37 homologs in the extracts of two other major STHs, whipworm (*Trichuris muris*) and hookworm (*N*. *americanus*, *A*. *ceylanicum*, and *A*. *caninum*) (Fig 2, left panel). Normal mouse sera at the same dilution did not recognize As37 and any of its homologs (Fig 2, right panel). Importantly, there was no immunological cross-reaction with human spleen tissue, despite the fact that this protein shares structural domains of the immunoglobulin superfamily (IgSF) in humans [38]. The anti-As37 sera did not recognize BSA negative control protein.

**Fig 3:**
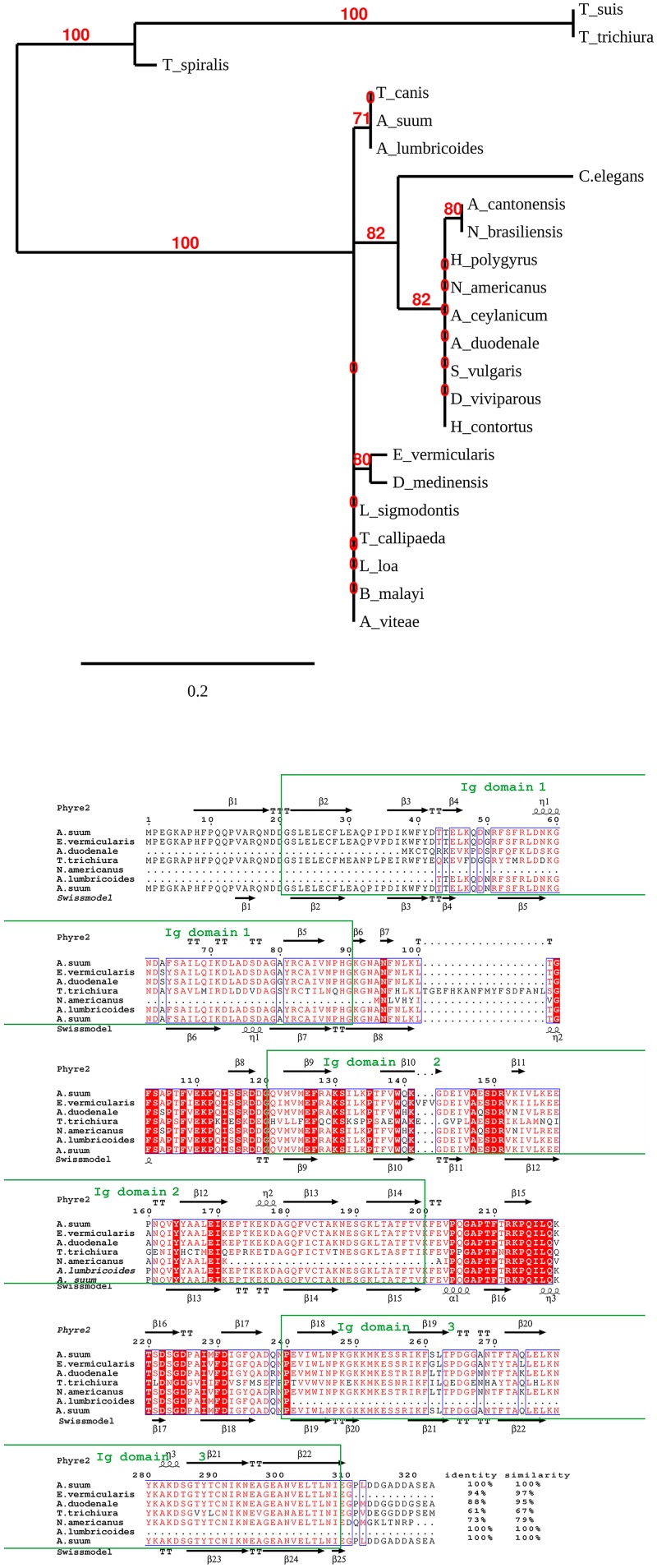
Sequence alignment of As37 with its homologs in other nematodes. (Top) Phylogenetic tree of As37 and its homologs in other nematodes, with branch support values in red. The sequences include As37 from *A*. *suum* (GenBank accession# BAC06575.1), *A*. *lumbricoides* (ALC00246), *Toxocara canis* (KHN88700.1), *Caenorhabditis elegans* (NP_001024421.1), *Angiostrongylus cantonensis* (KAE9417345.1), *Nippostrongylus brasiliensis* (VDL70373.1), *Heligmosomoides polygyrus* (VDP04139.1), *Necator americanus* (XP_013297867.1), *Ancylostoma ceylanicum* (EPB67396.1), *A*. *duodenale* (KIH68744.1), *Strongylus vulgaris* (VDM74829.1), *Dictyocaulus viviparous* (KJH47142.1), *Haemonchus contortus* (CDJ89388.1), *Brugia malayi* (XP_001899521.1), *Enterobius vermicularis* (VDD87484.1), *Dracunculus medinensis* (VDN56371.1), *Litomosoides sigmodontis* (VDM92971.1), *Thelazia callipaeda* (VDN01526.1), *Loa loa* (XP_020306393), *Brugia malayi* (XP_001899521.1), *Acanthocheilonema viteae* (VBB29563.1), *Trichinella spiralis* (XP_003369356.1), *T*. *trichiura* (CDW58363.1) and *T*. *suis* (KFD56086.1). (Bottom) Structure and amino acid sequence comparison of As37 with its homologs in common human soil-transmitted nematodes. The sequences were aligned with Clustal Omega and the secondary structural features are illustrated with the structural homology model of As37 using ESPript. Identical residues are shown in solid red, and conserved residues are in red. The secondary structure elements were generated by homology modeling with either Phyre2 or Swiss model, as indicated. The percentage of sequence identity or similarity to As37 is shown at the end of each sequence.

A BLAST search of GenBank revealed that As37 was a nematode-conserved protein identified in all nematode species with high sequence similarity (Fig 3 (top)). There was no homolog found in trematodes or cestodes. Importantly, the protein shares 100% sequence identity with its counterpart in the human roundworm *A*. *lumbricoides*, 94% identity to the human pinworm *Enterobius vermicularis*, 73% and 88% identity to the human hookworms *N*. *americanus* and *A*. *duodenale*, respectively, and 61% identity to the human whipworm *T*. *trichiura* (Fig 3 bottom). As37 also shares 99% sequence identity with its counterpart in the dog roundworm *Toxocara canis*. Structural and functional domain analysis revealed that As37 is a member of the immunoglobulin superfamily (IgSF) containing three immunoglobulin domains [39]. The predicted tertiary structure appears to be highly conserved among the select nematodes with 100% confidence in Phyre2, likely due to the three conserved Ig domains (labeled in green boxes). This high level of sequence and structure conservation among the common human STHs suggests immunization with a single As37-based vaccine or the homologs from other STHs would be suitable as a universal pan-helminth vaccine candidate inducing protective immunity against all STH infections.

### Immunization of rAs37 induces significant antigen-specific antibody responses

**Fig 4:**
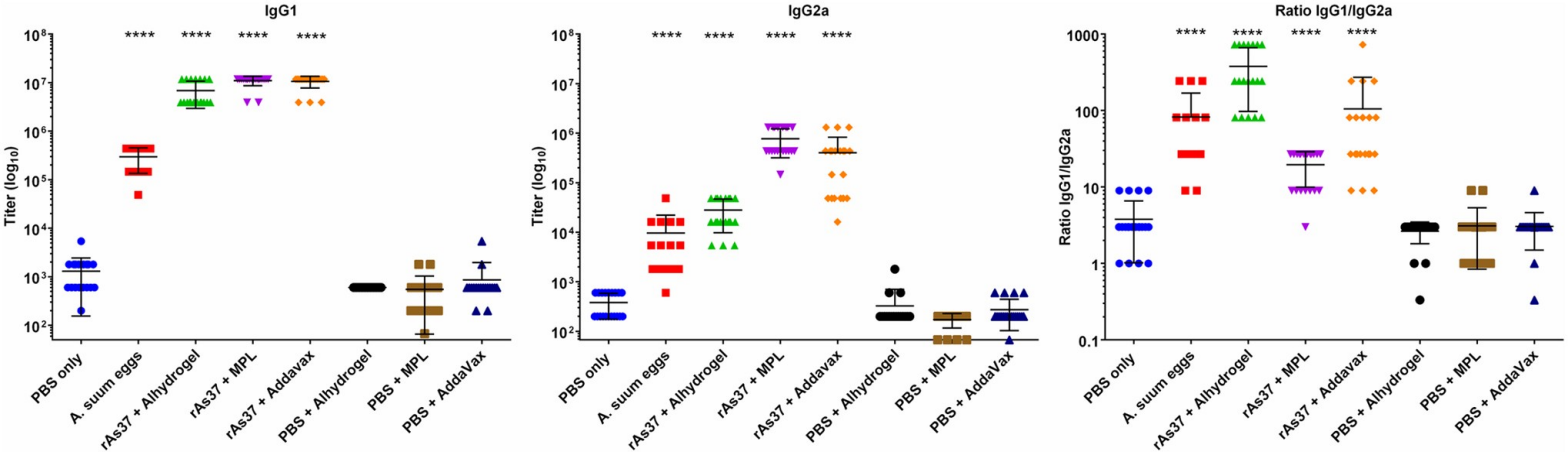
Anti-As37 IgG1and IgG2a titers (Log10) in sera of BALB/c mice immunized with rAS37 formulated with different adjuvants: Alhydrogel, MPL, and AddaVax, as measured by ELISA. Results are shown as means ± standard deviations and individual data points for each group (n = 15). The anti-As37 IgG1 and IgG2a titers were also measured in groups of mice infected with 1,000 *A*. *suum* infective eggs for three times. The anti-rAs37 IgG1/IgG2a ratio in each group was also shown. ****p< 0.0001.

Immunization with rAs37 formulated with Alhydrogel, MPLA, and AddaVax elicited significant-high titers of rAs37-specific IgG1 (up to 1x10^7^) in immunized mice. Immunization with rAs37 formulated with MPLA and AddaVax also induced significant IgG2a antibody responses, with the highest values in the MPLA group. The IgG1/IgG2a ratio was 380:1 in the Alhydrogel group, 105:1 in the AddaVax group and 20:1 in the MPLA group, emphasizing that Alhydrogel mainly induces a Th2-type response. MPLA induced the highest Th1-type response and AddaVax promotes a mixed Th1/2 type immune response [40] (Fig 4). Interestingly, repeated infections with *A*. *suum* infective eggs induced significantly high titers of anti-As37 IgG1 and IgG2a in mice, consistent with the Western blot results of rAs37 being recognized by the *A*. *suum* infected mouse sera in Fig 1. This further confirms that native As37 is exposed to the host immune system during the natural infection. As expected, mice receiving adjuvant only did not show significant IgG and IgG isotype responses to rAs37.

### Vaccination of rAs37 induces significant antigen-specific cytokine release

**Fig 5:**
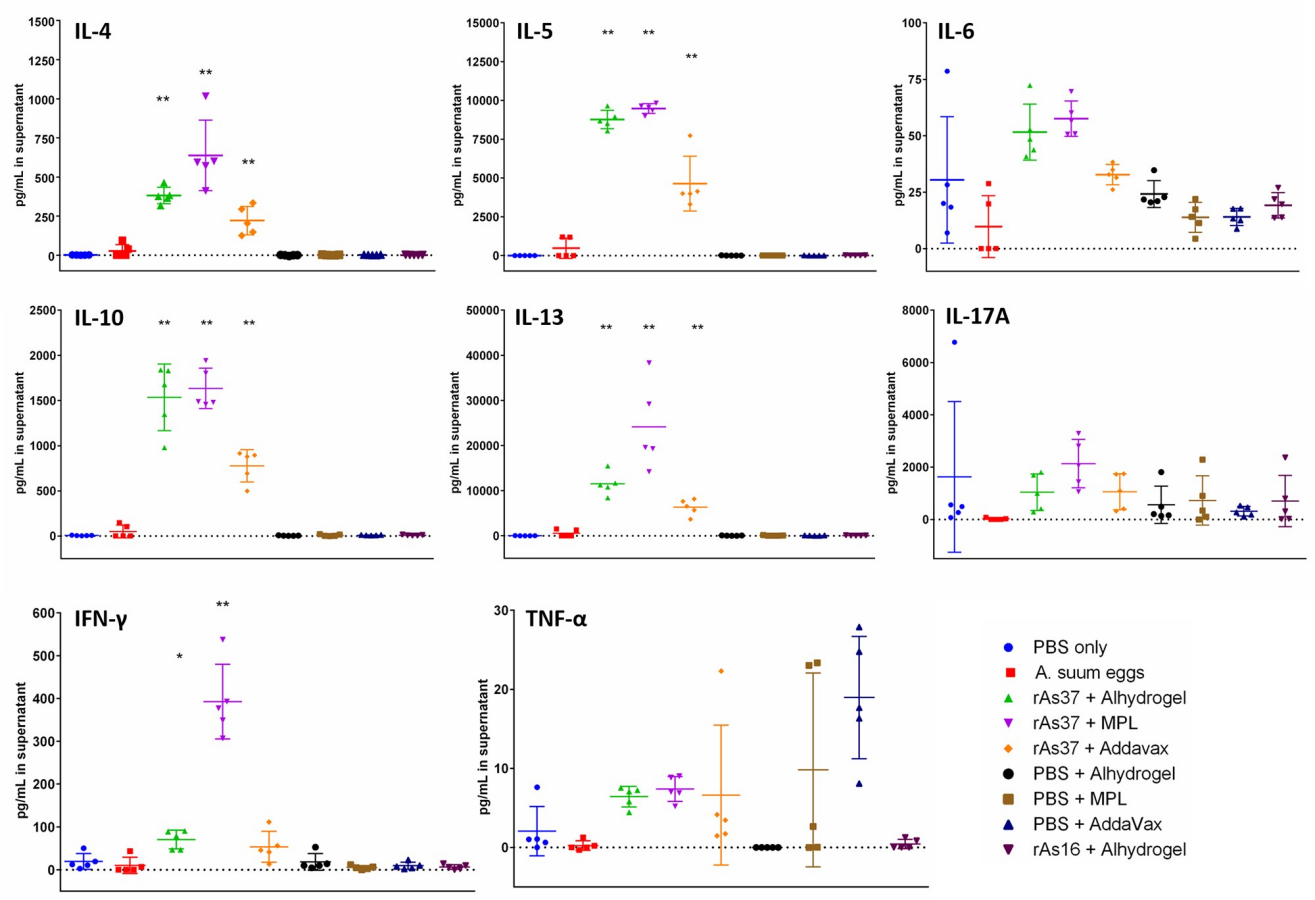
Cytokine profiles of BALB/c mice immunized with rAs37 formulated with Alhydrogel, MPL, and AddaVax. Cytokine levels were determined in supernatants of splenocytes after being re-stimulated with rAs37 (25 μg/ml) for 48 hours. Cytokine values measured in unstimulated samples were subtracted from the cytokine values from the corresponding rAs37 re-stimulated samples. Data are presented as means and individual values for each group (n = 5). *p< 0.05, **p< 0.01.

To evaluate the cellular response induced by immunization with rAs37 formulated with different adjuvants, the splenocytes were isolated from each immunized mouse and re-stimulated with 25 μg/ml rAs37. The cytokine measurement showed that the levels of IL-4, IL-5, IL-10 IL-13 in the supernatants of splenocytes collected from all mice immunized with rAs37 formulated with Alhydrogel, MPL, and AddaVax, were significantly elevated upon the re-stimulation with 25 μg/mL rAs37. IL-6 was also elevated in all As37 immunization groups, but not significantly compared to the PBS control. IFN-γ production was strongly stimulated in mice immunized with rAs37 formulated with MPL (*p*<0.01), slightly increased in the group immunized with rAs37 formulated with Alhydrogel (*p*<0.05). There was no significant response for IL-17a and TNF-α (Fig 5). Mice infected with *A*. *suum* infective eggs did not show detectable cytokine responses upon As37 stimulation, possibly the native As37 without an adjuvant only induces an antibody response. Splenocytes from the PBS or adjuvant control groups also did not show detectable cytokine expression.

### Vaccination of rAs37 induced protection against *A*. *suum* challenge

**Fig 6:**
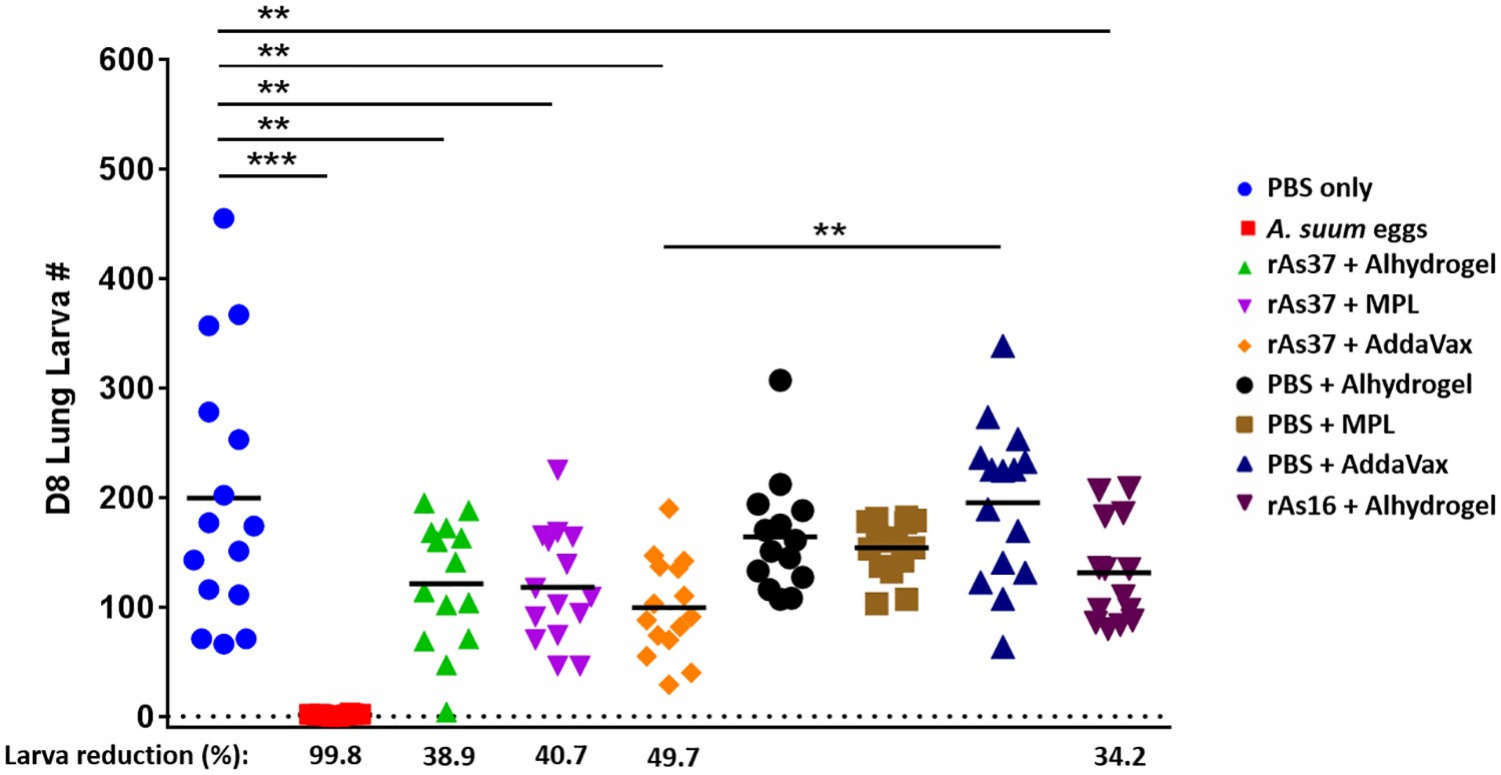
Lung larva reduction in mice immunized with rAs37 formulated with different adjuvants (Alhydrogel, MPLA, AddaVax) on Day 8 after being challenged with 2,500 *A*. *suum* eggs, presented as the mean ± SD (N = 15). L The asterisks indicate statistically significant differences in larval reduction compared to the PBS or adjuvant control groups (**p< 0.01, ***p<0.001).

Challenge study showed that mice immunized with 25 μg rAs37 formulated with AddaVax, Alhydrogel and MPLA produced significant larva reduction (49.7%, 38.9%, and 40.7%, respectively) against challenge with 2,500 *A*. *suum* infective eggs, compared with the group that received PBS only (*p*<0.01). The lung larvae reduction was also observed in rAs37 immunized mice when compared to the corresponding adjuvant control groups, however, only mice immunized with As37 formulated with AddaVax showed statistically significant lung larvae reduction (48.7%) compared to the AddaVax control group (*p*<0.01). The protection induced by rAs16 [25] was further confirmed in this study. Similar to our previous study, mice repeatedly infected with *A*. *suum* infective eggs acquired near sterile immunity in this trial (99.8% lung larva reduction) (Fig 6).

## Discussion

As37 is an immunodominant antigen exposed to the host immune system that induces strong immune responses during natural infection with *A*. *suum* eggs [21]. In this study, we evaluated and confirmed that an *E*. *coli* expressed As37 recombinant protein was strongly recognized by sera from *A*. *suum* infected mice that had gained sterile protection against re-infection with *A*. *suum* infective eggs (99.6%). Therefore, As37 could be one of the *A*. *suum* expressed antigens that induces protective immunity during *A*. *suum* larval migration and would be highly suitable as a potential vaccine candidate for preventing infection. In this study, the anti-rAs37 mouse sera were able to recognize native As37 in the extracts of *A*. *suum* larvae, but not in the ES products. These results are consistent with the original finding that As37 is a somatic protein present in muscle, hypodermis, uterus, eggs, and intestine of the female worm [21].

Sequence and structure analysis revealed that As37 contains three immunoglobulin domains and belongs to the immunoglobulin superfamily (IgSF). The IgSF domain comprises approximately 100 residues formed by two β-sheets packed face to face [41]. The IgSF is one of the largest families of proteins in the genome of multicellular eukaryotes including 80 IgSF in free-living nematode *Caenorhabditis elegans* and 142 in *Drosophila melanogaster* [42]. Those 80 IgSF proteins in *C*. *elegans* are classified into 6 clusters. The two largest clusters include 8 members of Zig proteins and five members of PVR-like kinases, the other four each have only two members. Only 22 out of the 80 *C*. *elegans* proteins have been previously identified through experiments [42]. Although the members of the IgSF are diverse, they are structurally grouped as only four “sets” of V, C1, C2 and I [39,43]. *C*. *elegans* IgSF members belong to the Set I subclass based on Hidden Markov modeling or specific I set key residue patterns in their sequences [41]. As37, like *C*. *elegans* IgSF member proteins, is classified as a Set I IgSF protein, which is highly conserved in other nematodes. It shares 61–100% sequence identity and structural similarity with other human STHs such as the roundworm *A*. *lumbricoides* (100%), pinworm *E*. *vermicularis* (94%), hookworms *N*. *americanus* (73%) and *A*. *duodenale* (88%), and whipworm *T*. *trichiura* (61%). Western blot analysis with an anti-As37 antibody also confirmed that homologous proteins in other nematodes (hookworms, *Trichuris*) could be recognized by the anti-As37 antibodies in this study.

Typically, members of the IgSF protein superfamily are located on the surface of cells with the extracellular regions containing one or more immunoglobulin domains. IgSF proteins have a variety of functions as cell adhesion molecules (CAMs) to regulate signaling or adhesion. With their involvement in the cell surface recognition they control the behavior of cells in various tissues [43], especially in the neuronal signal conduction and development including axon development and functions [44], neuronal migration, synapse formation and function [45], and cancer metastasis [46]. Some IgSF proteins use cis-interactions to form receptors or auxiliary subunits of such receptors on the extracellular surface for some secreted ligands or viruses [47]. These characteristics and functions of IgSF make it an important molecule or receptor for the regulation of signaling or adhesion of a variety of organisms. In addition to its role as an immunodominant protein expressed by *Ascaris* larvae, As37 was highly expressed on the surface of *A*. *suum* adult worms. These findings indicate As37 may be involved in the interaction of larval or adult worms with the host and may have an important role for the survival of the worm in the host [21].

Due to its immunodominance during *Ascaris* infection and recognition by protective immune sera, as well as its role as IgSF molecule, rAs37 was evaluated for its immunogenicity and vaccine efficacy. Mice were immunized subcutaneously with rAs37 formulated with different adjuvants, Alhydrogel, MPLA, and AddaVax, then challenged with *A*. *suum* embryonated infective eggs. The challenge results demonstrated that immunization with rAs37 formulated with different adjuvant produced a significant reduction of larvae migrated to lungs 8 days after infection, specifically 38.90% larval reduction when formulated with Alhydrogel, 40.7% with MPLA and 49.7% with AddaVax compared to the PBS control group (*P*<0.01). Another vaccine antigen rAs16 that induced protection in our previous study [25] was used as a positive control in this vaccine trial study and mice immunized with rAs16 formulated with Alhydrogel had 34.2% lung larval reduction (*P*<0.05). However, compared to each adjuvant alone control, only As37 formulated with AddaVax induced significant protection (48.7%, *P*<0.01). Alhydrogel is an alum-based adjuvant mainly stimulating a Th2-type response [48,49], confirmed by the high ratio of IgG1/IgG2a (380:1) and high levels of IL-4, IL-5, and IL-10 in this study. MPLA (Monophosphoryl Lipid A) is a potent activator of TLR4 that stimulates a strong Th1 response or a combined Th1/Th2 response [50], confirmed by high levels of INF-γ and a low IgG1/IgG2a ratio (20:1). We also observed the secretion of IL-5, IL-10, and IL-13 upon stimulation with rAs37 in this study. Mice immunized with rAs37 formulated with AddaVax showed strong IgG1 and IgG2a responses with an IgG1/IgG2a ratio of 105:1, which is lower than the ratio from the Alhydrogel group (380:1) but higher than that we observed with MPLA (20:1). In addition, we saw production of IL-4, 5, 10 and 13, further indicating a mixed and balanced Th1/Th2 response. The highest protection (lung larva reduction, 49.7%) was achieved by immunizing with rAs37 formulated with AddaVax; this is possibly due to a balanced Th1 and Th2 response that kills the infected larvae during their migration to the lung of infected mice. AddaVax is a squalene-based oil-in-water nano-emulsion adjuvant similar to MF59 that has been approved for human flu vaccines in Europe [51]. It has been proven to stimulate both cellular (Th1) and humoral (Th2) immune responses [52,53]. Immunization with AddaVax formulated rAs37 stimulated the secretion of IL-5 that could induce B cell differentiation and antibody secretion, and is a strong regulator of eosinophil formation, maturation, recruitment and survival [54], which has been determined important for the control of helminth infections [17]. The IL-5 induced eosinophil infiltration in the *A*. *suum* larva infected lungs can stimulate the differentiation maturation of antigen-specific T-cell [55,56], except for the direct kill by antibody-mediated ADCC or degranulation that releases antimicrobial cytotoxic or other molecules [57]. Some research has demonstrated that Th1 cytokine TNF-α combined with IL-13 was involved in the expulsion of *T*. *muris* [58–60]. The increased level of IL-13 was observed in this study with AddaVax as an adjuvant, however, the increase of TNFα was not significant.

In this study, we have identified for the first time that immunization with recombinant As37 formulated with AddaVax induced about 50% larval reduction in mice against a challenge with *A*. *suum* infective eggs. It is very common to face low worm reduction (30–50%) or non-sterilizing immunity induced by a single subunit vaccine in helminth infections [61], possibly because helminth parasites have complicated life cycles and, as multicellular organisms, present a large number of different antigens to the host immune system. The combination of multiple protective antigen as multivalent vaccine will therefore be a more promising approach to induce better protection. As37 and the previously identified protective antigen As16 will be used in our next vaccine efficacy test in order to induce higher protection against *Ascaris* infection.

We note that all vaccine efficacy tests were performed in a mouse model. Since mouse is not a permissive host for *A*. *suum* and adult worms cannot develop in mouse. The vaccine efficacy of As37 must thus be re-evaluated in a pig model. If the protection for the adult worm reduction is indeed confirmed in the pig model, there will also be great potential benefit for its use as a veterinary vaccine. *A*. *suum* infection in pigs is very common and causes big financial losses in the industry [15].

Sequence and homology analysis demonstrated that As37 is a member of the IgSF family and a nematode conserved protein. As37 shares 100% sequence identity with the human *A*. *lumbricoides* parasite as well as high homology with other soil-transmitted nematodes (hookworm, *T*. *trichiura* and *E*. *vermicularis*). Anti-As37 antibodies strongly recognized homologs from these nematodes but did not recognize human spleen tissue extracts. Even though more than 765 members of the IgSF family were identified in the human genome, the sequence homology to As37 is less than 23% [38], which indicates that there is a low probability of triggering immunological cross-recognition with human tissues. These results further suggest that the nematode-conserved As37 could be developed as a vaccine antigen to prevent all STH infections without reacting with human IgSF molecules. A pan-helminth vaccine is the ultimate goal to prevent and control STH infections in co-endemic areas and As37 could be a promising candidate towards this goal. In addition to its vaccine potential for pan-STH in human, As37 could be a good vaccine candidate for infection of *T*. *canis*, a common dog roundworm, due to its nearly identical sequence. Toxocariasis caused by larval migration through different tissues after ingestion of *T*. *canis* eggs is the most common helminth infection in the US [62]. Further cross-protection studies with As37 against other STH infections with different challenge models are underway.
